# The mechanisms of glycolipid metabolism disorder on vascular injury in type 2 diabetes

**DOI:** 10.3389/fphys.2022.952445

**Published:** 2022-08-31

**Authors:** Xiatian Chen, Chengzhen Shi, Yin Wang, Hua Yu, Yu Zhang, Jiaxuan Zhang, Peifeng Li, Jinning Gao

**Affiliations:** ^1^ Center for Molecular Genetics, Institute of Translational Medicine, The Affiliated Hospital of Qingdao University, Qingdao University, Qingdao, China; ^2^ School of Basic Medicine, Qingdao University, Qingdao, China; ^3^ Juxian People’s Hospital, Rizhao, China; ^4^ The Affiliated Cardiovascular Hospital of Qingdao University, Qingdao, China

**Keywords:** glucose metabolism, lipid metabolism, vascular injury, T2D, therapeutic strategy

## Abstract

Patients with diabetes have severe vascular complications, such as diabetic nephropathy, diabetic retinopathy, cardiovascular disease, and neuropathy. Devastating vascular complications lead to increased mortality, blindness, kidney failure, and decreased overall quality of life in people with type 2 diabetes (T2D). Glycolipid metabolism disorder plays a vital role in the vascular complications of T2D. However, the specific mechanism of action remains to be elucidated. In T2D patients, vascular damage begins to develop before insulin resistance and clinical diagnosis. Endothelial dysregulation is a significant cause of vascular complications and the early event of vascular injury. Hyperglycemia and hyperlipidemia can trigger inflammation and oxidative stress, which impair endothelial function. Furthermore, during the pathogenesis of T2D, epigenetic modifications are aberrant and activate various biological processes, resulting in endothelial dysregulation. In the present review, we provide an overview and discussion of the roles of hyperglycemia- and hyperlipidemia-induced endothelial dysfunction, inflammatory response, oxidative stress, and epigenetic modification in the pathogenesis of T2D. Understanding the connections of glucotoxicity and lipotoxicity with vascular injury may reveal a novel potential therapeutic target for diabetic vascular complications.

## 1 Introduction

**FIGURE 1:**
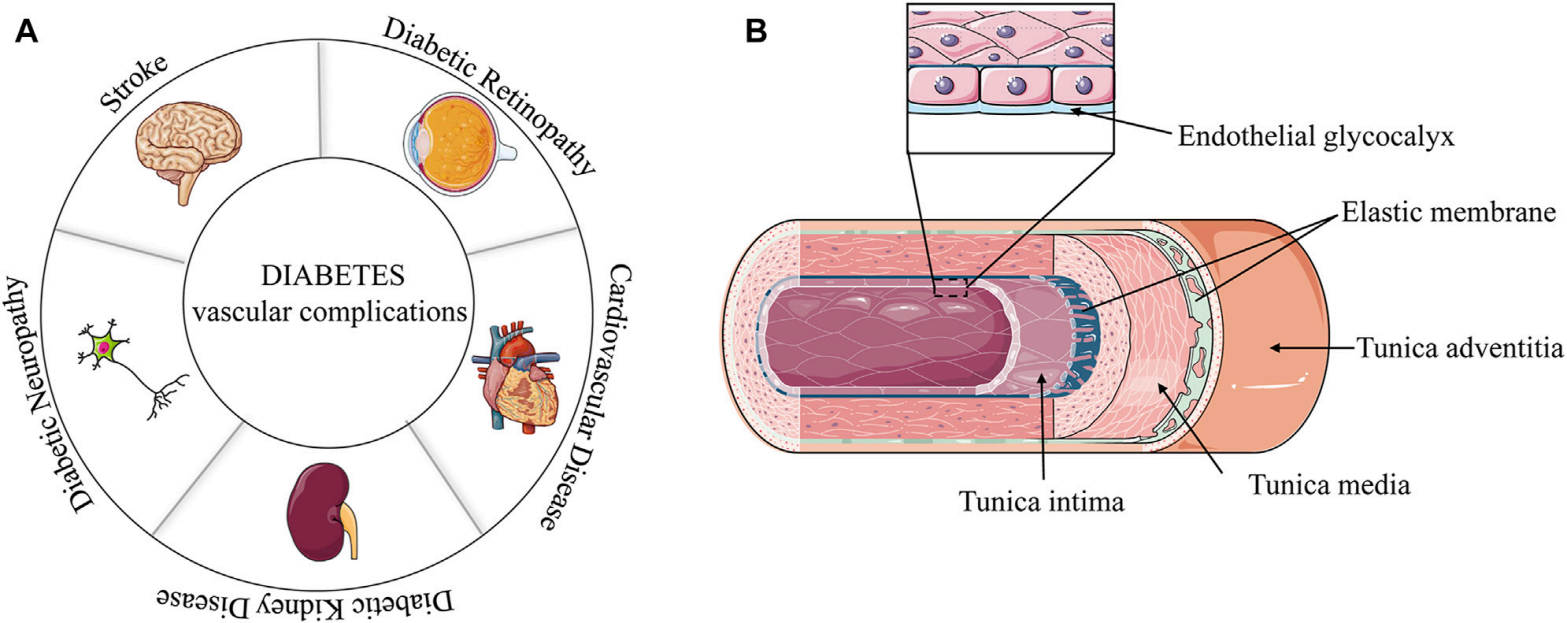
**(A)** Vascular complications of diabetes. **(B)** Diagram of a vascular structure.

Diabetes mellitus (DM) is a heterogeneous disease with multiple etiologies. Diabetes is more common in the middle-aged and elderly populations. However, the age of diabetes onset tends to be younger because of excess nutrition and lack of exercise. Currently, approximately 463 million people have diabetes worldwide (Ogurtsova et al., 2017). Diabetes is generally divided into type 1 diabetes (T1D) and type 2 diabetes (T2D) according to the function of human pancreatic islets, with T2D accounting for most cases (Beckman and Creager, 2016; Schwab et al., 2021; Chen et al., 2022). Insulin resistance is a significant feature of T2D. Unhealthy diet, obesity, and physical inactivity increase the risk of T2D (Teodoro et al., 2018; Georgakis et al., 2021). The T2D phenotype includes the inability to metabolize glucose in blood vessels, resulting in high glucose (HG) contents in blood vessels. Excessive lipid accumulation is associated with glucose intolerance (Nosadini and Tonolo, 2011). Dyslipidemia is another indicator of T2D, as it increases the low-density lipoprotein (LDL) concentration (Wils et al., 2017). Uncontrolled glycolipid metabolism in T2D can damage and destroy blood vessels in various organs, especially the kidneys, eyes, heart, and nerves (Iacobini et al., 2021). The vascular complications of T2D, such as diabetic retinopathy, diabetic nephropathy, diabetic neuropathy, myocardial infarction and stroke, are mostly related to the aforementioned tissues (Figure 1A).

The pathophysiology of the association between T2D and vascular complications is multifactorial. Several studies have revealed that it was closely related to glucotoxicity and lipotoxicity (Teodoro et al., 2018; Opazo-Ríos et al., 2020). Hyperglycemia and hyperlipidemia could trigger oxidative stress through mitochondrial dysfunction, and then enhance the production of reactive oxygen species (ROS), whereas hyperlipidemia releases pro-inflammatory cytokines through adipose tissue. Oxidative stress and inflammatory response are considered major factors in the progression of T2D and its complications. These lesions can manifest as endothelial dysfunction (ED), an early stage of vascular disease and T2D complications. Furthermore, epigenetic reprogramming can affect the genes associated with inflammation and the generation of ROS, resulting in ED. In the present work, we reviewed the blood vessel damage caused by abnormal glucose and lipid metabolism, which may provide insights for developing new strategies for the treatment of T2D and its complications.

## 2 Vascular wall structure

In general, blood vessels walls are divided into three layers from the lumen to the outside: the tunica intima, tunica media, and tunica adventitia (Jedlicka et al., 2020). The tunica intima is the innermost layer of the tube wall, which is mainly composed of interconnected endothelial cells. However, there are also gaps in some blood vessels, especially in the veins. The tunica media varied in composition and thickness according to the type of blood vessel. The aorta is mainly based on the elastic membrane, and the middle artery is mainly made up of smooth muscle. The tunica adventitia is mainly composed of fibroblasts, elastic fibers, and collagen fibers.

For a long time, the inner membrane cells have been generally believed to be the inner part directly in contact with blood. However, a later study found a colloidal film, called glycocalyx, that has a layer of protein-polysaccharide complex on the inner membrane cells and a thickness in the order of microns (Jedlicka et al., 2020). The vascular endothelial glycocalyx covers the surface of all vascular endothelial cells and regulates vascular endothelial permeability. The endothelial glycocalyx interacts with blood and endothelial cells, mediate blood flow shear force, and induce the release of nitric oxide (NO) (Becker et al., 2010; Becker et al., 2015; Jedlicka et al., 2020) (Figure 1B). Some blood vessels are also differently composed and distributed, and the veins have additional structures, such as venous valves.

## 3 Vascular damage due to hyperglycemia and dyslipidemia

**FIGURE 2:**
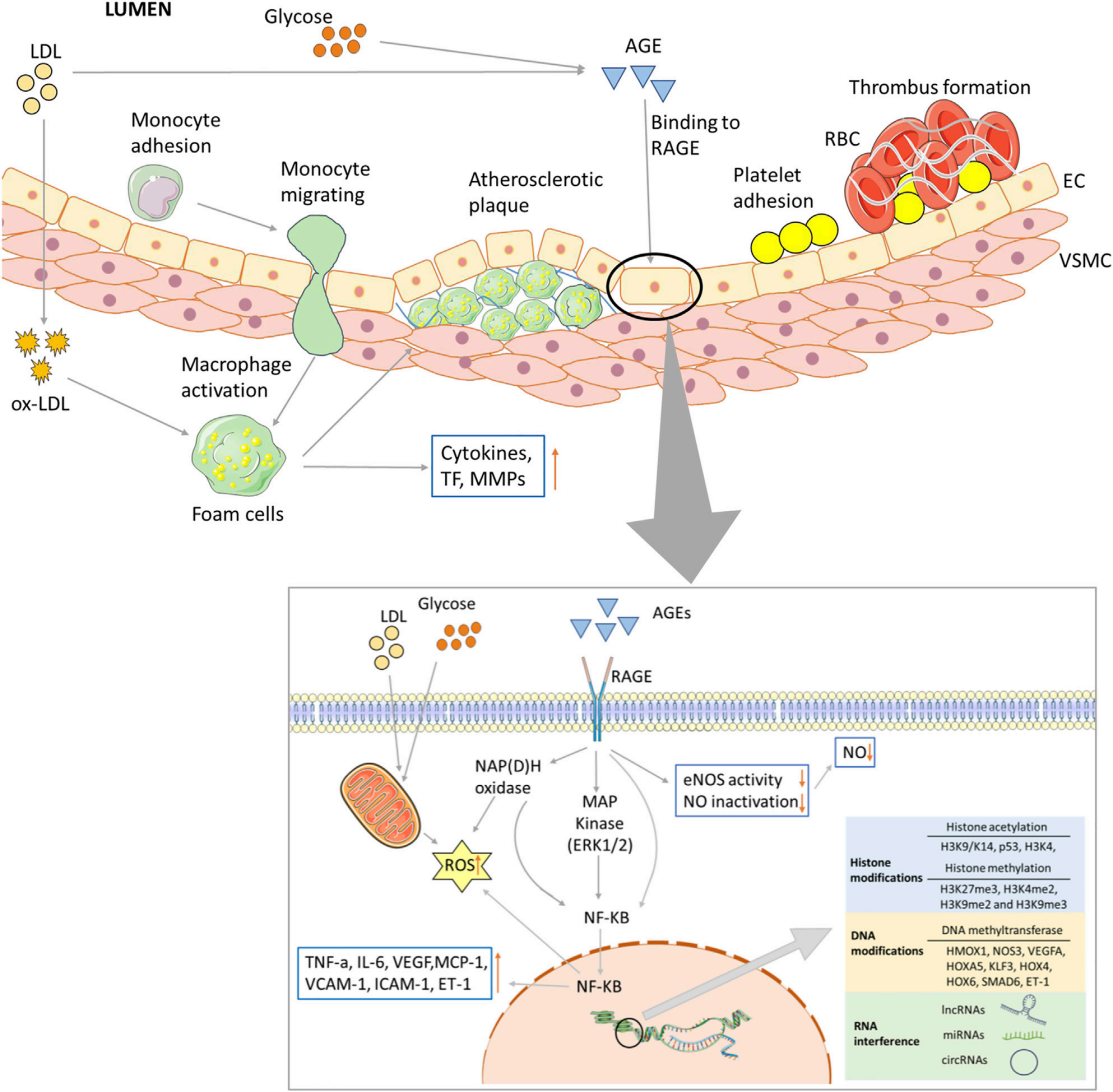
Biochemical and major pathways underlying endothelial dysregulation in vascular complications of diabetes. Long-term hyperglycemia and hyperlipidemia can cause endothelial cell dysfunction and increase the adhesion of monocytes and platelets. The former can transform into macrophages, while the latter can recruit blood cells, accumulate in blood vessels, and form thrombi. On the one hand, macrophages invade endothelial cells and engulf ox-LDL, turning into foam cells and forming arterial plaques. Macrophages release inflammatory and transcription factors that aggravate the inflammatory response. Excessive glucose and lipid levels will covalently combine to form AGEs, which can bind to their receptors; activate the MAPK and NF-KB pathways, among others; and reduce the production and utilization of NO. Abnormal glucose and lipid metabolism can also affect mitochondrial function, produce excessive ROS, and lead to insufficient energy supply. Epigenetic modifications are also closely related to vascular injury in the vascular complications of T2D, including histone and DNA modifications, and ncRNA regulation.

Hyperglycemia associated with dyslipidemia initiates diabetic vascular complications through metabolic and vascular remodeling and aberrant gene expression. In recent years, many hypotheses have been proposed to elucidate the mechanisms of hyperglycemia and dyslipidemia evoked vascular damage, including ED, the formation of advanced glycation end products (AGEs), oxidative stress, inflammation and epigenetic modification (Figure 2).

### 3.1 ED and structure remodeling

Endothelial cells not only constitute the vascular network but also are involved in vasodilation and vascular reactivity. The early sign of diabetic vascular injury is endothelial injury. Endothelial cells regulate the tension in blood vessels through mechanoreceptors, which sense shear forces caused by blood flow through their surfaces. Subsequently, endothelial cells transmit these messages to surrounding cells and secrete many biologically active mediators to dilate or contract blood vessels to regulate blood flow (Iliff and Xu, 2018). For example, the active mediators of vasodilation are NO, prostaglandin I2 (PGI2), histamine, and serotonin. The vasoconstrictive mediators are endothelin-1 (ET-1), thromboxane A2, angiotensin II (Ang II), and prostacyclin H2 (Sandoo et al., 2010; Krüger-Genge et al., 2019). HG and LDL levels can change the osmotic pressure of endothelial cells and the permeability of the intima, which may affect the synthesis and release of vascular regulatory factors by endothelial cells, resulting in reduced vascular reactivity.

The normal metabolism of intimal cells and the integrity of the intima are the basis for the normality of vessels. ED is the inability of endothelial cells to maintain vascular homeostasis. The development of ED is related to an increased risk of vascular complications and mortality (Erkens et al., 2017; Heusch, 2020). Oxidative stress has been considered a sign of ED. AGEs promotes cellular glucotoxicity, and long-term ED supports inflammation in the vascular system.

As mentioned earlier, NO is a vasodilator that can induce the relaxation of vascular smooth muscle cells (VSMCs) and vascular vasodilation (Moncada et al., 1991; Rajendran et al., 2019). ED can inhibit the expression of endothelial nitric oxide synthases (eNOS) and decrease NO synthesis. Glycocalyx is a protector of endothelial cells. However, HG levels could directly damage the glycocalyx by promoting the shedding (Jedlicka et al., 2020). Endothelial cells are sensitive to HG levels, and the change in the extracellular environment can induce cell death and influence cell growth (Triggle et al., 2012). The excess glucose can activate NF-kB expression in endothelial cells, which leads to an increase in the expression levels of pro-inflammatory cytokines (Aminzadeh et al., 2013). HG can stimulate the transformation of endothelial cells into fibrous phenotypes, causing vascular injury. Several studies have found fibrotic changes in the heart, liver, and kidneys of diabetic rodent models (Huang et al., 2013; Lucchesi et al., 2015; Alex et al., 2018). ED conditions can induce increased VSMC proliferation and phenotypic transition, increasing the thickness of the vascular middle layer and promoting the stenosis of the vascular lumen.

Oxidized low-density lipoprotein (ox-LDL) can be formed when LDL levels increases in blood vessels, and the lipid hydrogen peroxide produced in the process of ox-LDL production can directly damage endothelial cells, increase cell permeability, and promote cell death. Endothelial cells damage will further cause structure remodeling to blood vessels. Monocytes can migrate into the inner membrane when the tunica intima is incomplete. Macrophages converted from monocytes engulf ox-LDL and turn into foam cells. This will increase the thickness of the median-intimal membrane of blood vessels. In addition, ox-LDL can inhibit the synthesis of the endothelial cell-derived relaxation factor or NO, impairing the normal diastolic function of arterial walls.

### 3.2 Formation of AGEs

AGEs are compounds of non-enzymatic glycation. Excess glucose, lipids and proteins combine to form covalent add-ons (Barlovic et al., 2011; Yang et al., 2019a; Papachristoforou et al., 2020). The free amino group of proteins and the carbonyl group reduce glucose levels or another carbonyl to develop the Schiff bases. The Schiff bases could undergo Amadori rearrangement reactions and form relatively stable aldehyde and amine products. The Amadori products can result from dehydration, rearrangement and fragmentation reaction, which lead to the formation of AGEs. Even though the last reaction is usually slow, ROS and other catalysts can increase its rate.

The increased levels of AGEs were related to the less endothelial-dependent vasodilation. AGEs can depress the expression of eNOS by receptor-mediated phosphorylation of serine residues in eNOS and increase the degradation of eNOS mRNA (Ren et al., 2017). Tan et al. found that increased concentrations of AGEs were associated with ED by impairing endothelial-dependent vasodilation (Tan et al., 2002). AGEs are also responsible for increases in ET-1 levels and decreases in PGI2 levels, which lead to vasoconstriction (Santilli et al., 2015). AGEs were related to lipid accumulation in vessels. For example, the circulating AGEs can alter the structures of LDL and damage the mechanisms of LDL receptor-mediated cholesterol uptake (Ruiz et al., 2020). Furthermore, the interaction of AGE and LDL could induce pro-inflammatory cytokine production and affect the phagocytosis of macrophages and phenotypic conversion of smooth muscle cells (Iwashima et al., 2000; Barlovic et al., 2011; Byun et al., 2017).

The interaction of AGEs and their receptor (RAGE) plays a vital role in the AGEs that mediate vascular complications. The binding of AGEs to RAGE activates the transcription factor NF-kB which regulate multiple genes, such as ERK (extracellular signal-regulated kinase) 1/2 and the p38 MAP kinase (MAPK) pathway (Bierhaus et al., 2001; Chen et al., 2010a). These signaling pathways lead to an inflammatory response, including the aggregation of cell adhesion molecules. NF-kB regulates various cellular signaling cascades associated with ROS formation, including NAPDH oxidase and protein kinase C (PKC) (Coughlan et al., 2009; Chen et al., 2010a).

### 3.3 Oxidative stress

Oxidative stress occurs when reactive species are overproduced or when the antioxidant system is weakened. Reaction species are usually derived from oxygen, nitrogen, or sulfur elements, which generate ROS, reactive nitrogen (RNS), and reactive sulfur. ROS and RNS are the major sources of oxidative stress. The free radicals include the superoxide anion radical (O_2_^−^), hydroxyl free radical (OH·), hydrogen peroxide (H_2_O_2_) and peroxynitrite (ONOO^−^).

Many experimental and clinical studies have demonstrated that oxidative stress promotes the development of vascular complication. The mitochondrial overproduction of ROS was reported to be the primary origin of vascular damage in hyperglycemia induced diabetic complications (Nishikawa et al., 2000; Iacobini et al., 2021). In hyperglycemia, the flux and voltage gradient of the electron donors into the electron transport chain through the inner mitochondria membrane were increased owing to the increase in pyruvate generated. The electron transfer inside complex III in the electron transport chain is plugged when the voltage gradient reaches a critical point. The electrons return to coenzyme Q and transfer to O_2_, resulting in superoxide (O_2_^−^) (Brownlee, 2005). In addition, NADPH oxidases, xanthine oxidase, uncoupled nitric oxide synthase (NOS), cycloxygenase-2 (COX-2), and endoplasmic reticulum (ER) stress can also contribute to the production of superoxide. Excessive ROS in turn causes mitochondria dysfunction and affects the energy supply of cells. In addition, dysfunctional mitochondria act as a key regulator of inflammatory response, apoptosis, and ferroptosis (Rizwan et al., 2020). Many apoptosis-related factors and key proteins are located in the mitochondria, and mitochondrial dysfunction could activate many signaling pathways (Li et al., 2018) such as the AMP-dependent protein kinase (AMPK)/the mammalian target of rapamycin (mTOR) (Assaf et al., 2022) and phosphatidylinositol 3-kinase (PI3K)/MAPK pathways (Barber et al., 2021).

Oxidative stress can directly induce vascular injury. For instance, oxidative damage can rapidly decrease the glycocalyx level in vascular endothelial cells, reducing the integrity of the vascular wall. ROS are a main factor in the progression of several disease. ROS can inhibit the activity of eNOS, increase the pressure in the mitochondrial and endoplasmic reticulum in endothelial cells, which leads to ED. Hyperglycemia can also increase the ROS production through the polyol and hexosamine pathways and PKC activation. In addition, hyperglycemia can directly trigger the excessive production of ROS by activating the NF-kB pathway which affect the enzymatic cascades (Pitocco et al., 2013). The nuclear factor erythroid 2-related factor (Nrf2) is an important transcription factor that regulates intracellular oxidative stress and is a key regulator for maintaining intracellular redox homeostasis (Baird and Yamamoto, 2020). Zhang et al. (2016) demonstrated that hyperglycemia could inhibit the regulation of Nrf2 activity, which leads to an imbalance in oxidative stress and the aggravation of cellular damage.

The excessive accumulation of ROS promotes the production of ox-LDL and AGEs, which can lead to vascular damage. However, ROS could oxidize lipids, proteins and nucleic acid molecules, including DNA and RNA. Studies have demonstrated that the oxidized nucleic acid content was higher in the urine or blood plasma of patients with T2D (Franzke et al., 2018; Schöttker et al., 2020). ROS can cause the DNA black, which leads to the activation of poly ADP-ribose polymerase (PARP), inhibiting the expression of the glycolytic enzyme glyceraldehyde-3-phosphate dehydrogenase (GAPDH), and promoting the production of early glycolytic intermediates (Shah and Brownlee, 2016). This production promotes ED through the PKC pathway and the formation of the AGE pathway. The oxidization of DNA can affect the its self-reparative ability and hyperglycemia disturbs the self-reparative ability of DNA by ROS, which leads to the cell death and senescence. A previous study revealed that DNA repair contributes to fibrotic remodeling (Kumar et al., 2020). Oxidative DNA can activate the homeo-domain interacting protein kinase 2 (HIPK2) expression, which leads to activation of fibrosis and then increase vascular stiffness (Tuleta and Frangogiannis, 2021).

RAGE is also a critical factor in oxidative stress-induced vascular damage. Many intracellular signaling pathway linked to oxidative stress can be activate when AGEs bind to RAGE. Furthermore, ROS can promote inflammatory response by increasing pro-inflammatory cytokine levels, the expression level of cell adhesion molecules, and growth factors. ROS can also activate multiple signaling molecules such as MAPK, PI3K, Akt (or PKB), mTOR, and JUK. These genes were reported to be associated with cell apoptosis, proliferation, migration, inflammatory response and oxidative stress (Chiarugi and Cirri, 2003; Torres and Forman, 2003; Montezano et al., 2014). Thus, the oxidative stress and inflammatory response mechanism were exacerbated, increasing endothelial cell apoptosis, inhibiting cell proliferation and vascular repair, and aggravating vascular injury.

### 3.4 Inflammation

Long-term chronic inflammation plays a major role in the pathogenesis and development of diabetic vascular complication, such as diabetic nephropathy and retinopathy. The inflammatory response in blood vessels can be stimulated by the aggregation of monocytes, platelets and the invasion of macrophages. The immune system is associated with the metabolic changes in T2D. Inflammation signaling is coupled with ROS and AGEs. Chronic inflammation can damage vascular components and the vascular endothelial structure and reduce vascular reactivity. The inflammatory reaction in the body directly destroys the glycocalyx, which is also a main hazard of long-term inflammation. Platelets, blood cells, and fibrin are deposited and aggregated to form thrombosis, damaging the endothelial cells in the intima, which recruit white blood cells and further activate the inflammatory response process. The inflammatory cells infiltrate the blood vessel wall, which can impair reactive and increase the stiffness of the blood vessel wall.

In T2D, the activation of inflammation is associated with dysregulated inflammasome. Plasminogen activator inhibitor type-1 (PAI-1) expression was associated with fibrinolysis and high PAI-1 expression is linked with formation of thrombosis. Pandolfi et al. found that PAI-1 levels were increased in the arterial wall of diabetic patients (Pandolfi et al., 2001). Evaluating PAI-1 expression level can help reduce fibrinolysis. HG levels can stimulate the accumulation of NLR family pyrin domain containing 3, resulting in the generation of interleukin-1β (IL-1β) (Sharma et al., 2018). The release of active IL-1β can induce the expression of other inflammatory cytokines and promote chemotactic responses and recruitment of macrophages.

Ox-LDL can enhance the expression of intercellular adhesion molecule-1, which increases the numbers of monocytes, neutrophils and lymphocytes binding to the endothelium, and the binding shows high affinity. Monocytes are transformed into macrophages and even induce ED and structural remodeling. This process can trigger ROS production, which promotes the expressions of IL-1 and tumor necrosis factor α (TNF-α). The increased IL-1 and TNF-α levels upregulated the expression of adhesion molecules and recruited more monocytes into the progression of vascular stiffness. These reactions can induce apoptosis and impair NO release, which leads to ED.

### 3.5 Epigenetic modification

Epigenetic modifications are stable and heritable changes to epigenetic inheritance and phenotypes, which are independent of changes in the gene sequence. Epigenetic factors could regulate gene expression and control cell phenotypes, including DNA methylation, histone modification and regulation of non-coding RNAs (ncRNAs). In T2D, abnormal regulation of enzymes in the gene promoter region could alter gene expressions, and differentially expressed genes such as SET domain-containing lysine methyltransferase 7 (SET7) and suppressor of variegation 3–9 homolog 1 (SUV39h1) can affect inflammation-related pathways that lead to vascular injury. Various genes can be changed by epigenetic modifications, which are involved in the pathology of diabetic vascular complications (Pirola et al., 2010; Kato and Natarajan, 2014). Those genes are mainly involved in the signaling of inflammation, modification, and oxidative stress.

#### 3.5.1 DNA methylation

DNA methylation refers to the covalent binding of a methyl group to the CpG dinucleotide cytosine 5′ carbon site in the genome without altering the DNA sequence by DNA methyltransferases (DNMTs) (Greenberg and Bourc’his, 2019). Previous studies have demonstrated that DNA methylation is involve in the progress of vascular complications (Rajasekar et al., 2015; Natarajan, 2021). The DNMT family includes DNMT1, DNMT2, and DNMT3 (DNMT3A, DNMT3B, and DNMT3L).

Hyperglycemia can stimulate DNA methylation. For instance, Priola et al. found the hypomethylation of heme oxygenase 1 (HMOX1) and hypermethylation of interleukin 8 precursor (IL8) in human aortic endothelial cells treated with HG (Pirola et al., 2011). Hyperglycemia can cause abnormal DNA methylation in multiple genes such as endothelial NOS, vascular endothelial growth factor A (VEGFA), homeobox A5, Krüppel-like factor 3, homeobox 4, homeobox 6, small mothers against decapentaplegic homolog 6 (SMAD6), and SMAD3, which are involved in angiogenesis, inflammation and migration resulting in ED (Dunn et al., 2015; Aref-Eshghi et al., 2020; Pepin et al., 2021). Another study also found that ET-1 was hypomethylated at the promoter region and during the worsened state of a vascular complication (Biswas et al., 2018).

However, hyperglycemia can also decrease DNA methylation. Mitochondrial adaptor p66^shc^ protein functions as a redox enzyme to regulate ROS generation and the oxidative signals associated with apoptosis. Paneni et al. (2012) found that hyperglycemia decreased DNA methylation at the promoter region of p66^shc^. Decreased methylation and upregulated p66^shc^ increased the AGEs precursors and promoted apoptosis. In the diabetic condition, the promoter of polymerase gamma 1 is hypermethylated in retinal endothelial cells (Tewari et al., 2012).

#### 3.5.2 Histone modification

Histones are proteins that make up nucleosomes together with DNA, including H2A, H2B, H3, and H4. Histone modification occurs covalently in the N-terminal tail part of the histone, and H3 is the most modified histone protein (Zhang et al., 2021a). These protein modifications can inhibit or activate the transcription of genes. The sites of histone methylation were usually lysine and arginine.

Histone acetylation is mainly related to the activation of genes, which are coordinated by acetyltransferase and deacetylase. For instance, H3K9/K14 acetylation was caused by HG level, which leads to the overexpression of IL-8, HMOX1, and MMP10. The upregulated genes were associated with oxidative stress and inflammation, which could promote the pathological process of T2D vascular complications (Pirola et al., 2011). The expression of sirtuin 1 (SIRT1) was negatively regulated by acetylated p53 under the HG condition. Upregulated SIRT1 inhibited the expression of PARP and mitochondria mediated apoptosis related genes, such as NF-kB and BAX (Orimo et al., 2009; Zheng et al., 2012). Overexpression of SIRT1 also restrained the expression of p66^shc^ and oxidative stress in the vasculature of diabetic mice (Zhou et al., 2011). Chen et al. (2010b) found that hyperglycemia can induce the expression of transcriptional coactivator p300 in human umbilical vein endothelial cells (HUVECs). Increased binding of p300 to ET-1 and fibronectin promoters, increased histone acetylation, H2AX phosphorylation, multi-transcription factor activation, expressions of vasoactive factors, and extracellular matrix protein. In the streptozotocin induced diabetic rat model, the histone acetyltransferases level was decreased and the histone deacetylases level was increased in HG levels (Zhong and Kowluru, 2010). Hyperglycemia triggered the expression of NF-kB, which is associated with increased H3K4 and reduced H3K9 methylation in aortic endothelial cells (Brasacchio et al., 2009).

H3K27me3 in the promoter of eNOS can regulate the expression and affect endothelial reactivity (Dhawan et al., 2022). Liao et al. (2018) found that histone methylation (H3K4me2, H3K9me2, and H3K9me3) at the promoter site reduced the expression levels of NOX4 and eNOS. Dysregulated NOX4 and eNOS contribute to ROS and NO generation, resulting in ED. Zhong et al. found that hyperglycemia reduced the expression of H3K4me1 and me2 but increased the binding of lysine-specific demethylase 1 (LSD1) with superoxide dismutase 2 (SOD2). Downregulation of the expression of LSD1 ameliorates the glucose-induced reduction of SOD2 H3K4 methylation and prevents the decrease in SOD2 gene expression levels (Zhong and Kowluru, 2011; Zhong and Kowluru, 2013). Hyperglycemia can promote the accumulation of SET7 and activate the activity of SET7. SET7 acts on the p65 promoter region of NF-kB, which leads to H3K4 methylation and upregulation of NF-kB expression and affects its dependent inflammatory factors such as the vascular cell adhesion molecule and monocyte chemotactic protein 1 (MCP-1). Previous studies have shown that SET7 knockdown can significantly reduce NF-kB-mediated inflammatory genes in diabetic models (Miao et al., 2006; Li et al., 2008). The increased expression levels of inflammation-related genes are also associated with decreases in the expression levels of repressive regulators in T2D. For example, at the promoter regions of IL-6, MCP-1, and TNF-α, decrease H3K9me3 and SUV39h1 recruitment were found in diabetic mice (Villeneuve et al., 2008). Furthermore, inhibition of the expression of SUV39h1 methyltransferase can reduce H3K9me3 expression in IL-6, which leads to cytokine expression (Villeneuve et al., 2010).

#### 3.5.3 ncRNAs

ncRNAs are transcribed from the genome but does not code proteins. They are involved in translation and regulate the expression of related genes. With the advancements of the sequencing technology and research, the understanding of non-coding RNA has also changed from “useless” to “useful.” Recent reports have implicated ncRNAs in the vascular injury in T2D, including microRNAs (miRNAs), circular RNAs (circRNAs), and long non-coding RNAs (lncRNAs; Table 1).

**TABLE 1:** ncRNAs associated with vascular dysfunction in diabetic patients and models.

	Model	Signaling	Function	Refs
miRNAs				
miR-34a	diabetic mouse/HUVEC	Sirt1	oxidative stress	Li et al. (2016)
miR-146a	diabetic rat	NF-kB	inflammation, apoptosis	Yousefzadeh et al. (2015), Habibi et al. (2016)
miR-221/222	diabetic mouse/VSMC	ERK-1/2/p27Kip1	proliferation, migration	Coleman et al. (2013), Lightell et al. (2018)
miR-342-3p	HUVEC/HDF	FGF11	proliferation, migration	Cheng et al. (2018)
miR-29	T2D subjects/diabetic rat	Lypla1	vasodilation	Widlansky et al. (2018)
miR-9	HUVEC	NICD1	inflammation	Chen et al. (2021)
miR-24	VSMC	PDGF-BB	inflammation, remodeling	Yang et al. (2018)
miR-19a	T2D subjects/HMEC	TF	inflammation	Witkowskiet al. (2018)
miR-483-3p	T2D subjects/HAEC	VEZF1	apoptosis, inflammation	Kuschnerus et al. (2019)
miR-210	T2D subjects/diabetic mouse	PTP1B	oxidative stress	Zhou et al. (2022)
miR-29c	diabetic mouse/VSMC	Emp2	proliferation	Torella et al. (2018)
miR-204	diabetic mouse/VSMC	Cav1	proliferation	Torella et al. (2018)
miR-181c-3p/-5p	T2D subjects/HUVEC	LIF	oxidative stress	Shen et al. (2018)
circRNAs				
circ_WDR77	VSMC	miR-124/FGF-2	proliferation, migration	Chen et al. (2017)
circHIPK3	HAEC/HUVEC	miR-124	death, apoptosis	Cao et al. (2018)
circDiaph3	VSMC	miR-148a-5p/IGF1R	proliferation, migration	Xu et al. (2019a)
circBPTF	HUVEC	miR-384/LIN28B	inflammation, oxidative stress	Zhang and Sui (2020)
circ_001209	diabetic mouse/HRVEC	miR-15b-5p/COL12A1	invasion, migration, tube formation	Wang and Zhang (2021)
circ_CLASP2	HUVEC	miR-140-5p/FBXW7	proliferation	Zhang et al. (2021b)
circSOD2	VSMC	miR-206/NOTCH3	proliferation, neointima formation	Mei et al. (2021)
circMAP3K5	SMC	miR-22-3p/TET2	differentiation, neointima formation	Zeng et al. (2021)
circ_0007367	PMVEC	NF-kB	inflammation, endothelial integrity	Li et al. (2022)
circ_0068087	PMVEC	miR-186-5p/ROBO1	inflammation, oxidative stress	Li et al. (2021a)
circ_0068087	HUVEC	miR-197/TLR4	inflammation	Cheng et al. (2019)
circ_0006768	HBMEC	miR-222-3p/VEZF1	migration, tube formation	Li et al. (2021b)
circ_0003423	BMEC	miR-589-5p/TET2	Endothelial injury	Yu et al. (2021)
circ_0003645	HUVEC	NK-kB	inflammation	Qin et al. (2020)
circ_0003204	HUVEC	miR-942-5p/HDAC9	oxidative stress, inflammation	Wan et al. (2021)
circ_36781	atherosclerosis mice/MAEC	miR-30days-3p/TP53RK	endothelial injury	Li et al. (2021c)
circ_37699	atherosclerosis mice/MAEC	miR-140-3p/MKK6	endothelial injury	Li et al. (2021c)
lncRNAs				
MALAT1	HBMEC	miR-126/PI3K/Akt	proliferation, angiogenesis	Zhang et al. (2020a)
MALAT1	HBMEC	miR-205-5p/VEGFA	proliferation, angiogenesis	Gao et al. (2020)
TUG1	HUVEC	Runx2/ANPEP	proliferation, migration	Du et al. (2021)
PVT1	HUVEC	miR-153-3p/GRB2	inflammation, oxidative stress	Guo et al. (2021)
H19	HUVEC	miR-let-7/periostin	Inflammation	Cao et al. (2019)
DANCR	VSMC/HUVEC	miR-214-5p/COX20	apoptosis	Zhang et al. (2022)
LINC00299	VSMC	miR-135a-5p/XBP1	apoptosis	Chang et al. (2022)
ZEB1-AS1	HUVEC	NOD2	proliferation, apoptosis	Xu et al. (2019b)
OIP5-AS1	HUVEC	miR-320a/LOX1	apoptosis	Zhang et al. (2020b)

##### 3.5.3.1 miRNAs

MiRNAs are small-molecule ncRNAs, with a length of 21–24 nucleotides, which function by partial complementary sequence binding to the 3′ untranslated region of the target mRNA to cleat the mRNA or repress the translation process (Ha and Kim, 2014). Each miRNA can have multiple target genes, and multiple miRNAs can regulate the same gene.

Silambarasan et al. (2016) performed a microarray on HUVECs treated with HG and analyzed in patients with T2D and diabetic rats. They found that the expression levels of ten miRNAs were gradually increased with the increase in HG concentration. Among these miRNAs, miR-29b-3p, miR-29c-3p, miR-125b-1-3p, miR-130b-3p, miR-221-3p, miR-320a and miR-192-5p were correlated with endothelial cell apoptosis. The expression of miR-34a was upregulated in the aortic endothelium of diabetic mice. MiR-34a knockdown can attenuate oxidative stress by regulating the expression of sirtuin1 (Sirt1) (Li et al., 2016). The low expression of miR-146a was associated with the significant increase in NF-kB expression level in diabetic rats, activated the inflammation pathway, and promote apoptosis (Yousefzadeh et al., 2015; Habibi et al., 2016). MiR-221 and miR-222 have a similar function, which is to promote the intimal thickening in the arteries of diabetic subjects. The downregulation of miR-221 and miR-222 reduced the VSMC proliferation and migration by metformin, and inhibiting miR-221 and miR-222 was efficacious in the prevention of the vascular complications of T2D (Coleman et al., 2013; Lightell et al., 2018). The miR-342-3p expression was downregulated in endothelial cells isolated from diabetic models. Overexpression of miR-342-3p enhanced endothelial cell proliferation and migration by targeting fibroblast growth factor 11 signaling (Cheng et al., 2018).

MiR-29a/b knockdown could impair the endothelial function in diabetic patients and rat models. Overexpression of miR-29 can promote NO production and restore endothelium-dependent vasodilation by modulating lysophospholipase I (Lypla1) expression (Widlansky et al., 2018). MiR-9 expression was involved in anti-inflammation and apoptosis under HG conditions (Jeyabal et al., 2016). Chen et al. (2021) found that upregulating the miR-9 expression can rescue hyperglycemia-induced ED by inhibiting the Notch1 signaling pathway. Overexpression of miR-24 can inhibit the platelet-derived growth factor pathway, protect against intimal hyperplasia and inhibit intracellular inflammatory responses (Yang et al., 2018). Endothelial injury stimulates platelet aggregation and cell proliferation and differentiation, and activated inflammatory signals can induce thrombosis. Previous studies have shown that the expression of miR-19a can regulate endothelial cell homeostasis and angiogenesis (Qin et al., 2010; Jiang et al., 2015). In addition, Witkowski et al. found that miR-19a expression correlated with miR-126 expression in plasma of diabetic patients and showed anti-thrombotic properties by modulating vascular tissue factor (TF) expression (Witkowski et al., 2018). Other study demonstrated that miR-483-3p could also regulate the endothelial integrity by targeting vascular endothelial zinc finger 1 (VEZF1) (Kuschnerus et al., 2019). Zhou et al. found that downregulation of miR-210 expression induced ED in T2D and the levels of miR-210 were lower in T2D patients. Overexpressed miR-210 can rescue endothelial apoptosis by repressing the expression of protein tyrosine phosphatase 1B (PTP1B) and oxidative stress (Zhou et al., 2022). Torella et al. found that miR-29c and miR-204 can regulate the VSMC hyperplastic phenotype by targeting epithelial membrane protein 2 (Emp2) and caveolin1 (Cav1), respectively (Torella et al., 2018). The expression of miR-181c is associated with endothelial cells damage under abnormal conditions of glycolipid metabolism (Yang et al., 2017). Shen et al. investigated whether miR-181c-3p/5p overexpression could enhance endothelial cell injury by regulating the expression of the leukemia inhibitory factor (Shen et al., 2018).

##### 3.5.3.2 circRNAs

CircRNAs are cyclic ncRNAs produced by non-canonical cleavage and covalently linked upstream and downstream shear sites (Kristensen et al., 2019). Many circRNAs have been identified in eukaryotes by high-throughput RNA sequencing (RNA-seq) and bioinformatics and were found to have tissue-specific expression patterns. circRNAs could act as miRNA sponges to affect the regulation of or interact with related proteins (Kristensen et al., 2019; Zang et al., 2020). To date, circRNAs have been reported to be associated with the pathologies and development of various diseases such as DM, cardiovascular diseases and cancer (Cortés-López et al., 2018; Altesha et al., 2019).

Vascular damage and repair can stimulate VSMC differentiation and proliferation, causing intravascular restenosis. Another reaction to vascular injury is intimal hyperplasia. Chen et al. (2017) performed a microarray and found 983 differentially expressed circRNAs under HG conditions. CircWDR77 (circ_0013509) expression was upregulated in VSMCs cultured with HG. circWDR77 knockdown inhibited cell proliferation and migration by targeting miR-124/growth factor 2 (FGF-2). CircHIPK3 expression was downregulated in HG-treated HUVECs and human aortic endothelial cells. circHIPK3 knockdown exacerbated endothelial cell apoptosis by modulating miR-124 expression (Cao et al., 2018). In the carotid artery injury rat model, overexpressed circDiaph3 (circ_005717) can act as a sponge with miR-148-5p and weaken the inhibitory effect of miR-148a-5p on insulin-like growth factor-1 receptor (IGF1R), which promotes intimal hyperplasia (Xu et al., 2019a).

A previous study found that circBPTF (circ_0045462) expression was upregulated in HUVEC under HG condition (Jin et al., 2019). Zhang and Sui, 2020 revealed that circBPTF knockdown could suppress cell apoptosis and reduce the release of pro-inflammatory cytokines by mediating the miR-384/LIN28B axis. Wang and Zhang, 2021 found that the circ_001209/miR-15b-5p/COL12A1 axis may be the potential regulatory pathway for human retinal vascular endothelial cells. Circ_CLASP2 (circ_0064772) expression was found to be downregulated in HG-induced HUVECs. Overexpression of circ_CLASP2 can decrease ED under HG conditions by inhibiting the expression of miR-140-5p and regulating FBXW7 expression (Zhang et al., 2021b). Depletion of circ_SOD2 alleviated VSMC proliferation by modulating the miR-206/NOTCH3 axis (Mei et al., 2021). Furthermore, Zeng et al. revealed that overexpression of circMAP3K5 was associated with reduced proliferation of VSMCs to sequester miR-22-3p, which inhibited the expression of TET2 (Zeng et al., 2021).

Flow pattern was related to endothelial integrity. Overexpression of circ_0007367 can protect the endothelial integrity by repressing the activity of NF-kB signaling and increasing the eNOS expression level (Li et al., 2022). The circ_0068087 expression level was increased in blood sample from patients with T2D. Circ_0068087 knockdown could ameliorate ox-LDL-induced HUVEC dysfunction by miR-186-5p and roundabout guidance receptor 1 (ROBO1) (Li et al., 2021a). Moreover, circ_0068087 knockdown could also suppress cell dysfunction under HG conditions. Inhibiting miR-197 expression can reverse the circ_0068087 function, and toll-like receptor 4 (TLR4) was the downstream target of miR-197 (Cheng et al., 2019). Li et al. (2021b) found that upregulated circ_0006768 expression rescued human brain microvascular endothelial cells injury by modulating the expression of miR-222-3p and its target, VEZF1.

Circ_0003423 functions as an endogenous miR-589-5p sponge to inhibit miR-589-5p activity that upregulates TET2, which relieves ox-LDL-induced endothelial cell injury (Yu et al., 2021). The circ_0003645 expression was found to be up-regulated in patients with atherosclerosis and *in vitro*. Cell apoptosis and the expression levels of the NF-kB pathway-related genes were reduced after knockdown of circ_0003645 expression (Qin et al., 2020). Changes in circ_0003204 levels have been detected in HUVECs (Liu et al., 2020; Wan et al., 2021). Silencing circ_0003204 promoted cell viability and decreased inflammation in HUVECs. Circ_0003204 can modulate HDAC9 levels by sponging miR-942-5p. Li et al. (2021c) performed a circRNA microarray to detect aberrant expression of circRNAs in an atherosclerosis mouse model. Finally, the circABCA1 (circ_36781) and circKHDRBS1 (circRNA_37699) expression were significantly upregulated. The following sponging of miR-30 days-3p and miR-140-3p were predicted using bioinformatics analysis, and the target protein TP53RK and MKK6 were identified *in vivo* and vitro, respectively.

##### 3.5.3.3 lncRNAs

LncRNAs are a diverse class of RNAs with lengths of more than 200 nucleotides. Studies have proposed many functions for lncRNAs, including cis or trans transcription regulation, nuclear domain organization, and interaction with other ncRNAs or specific proteins (Quinn and Chang, 2016). Previous studies have implicated lncRNAs in the progression of numerous pathologies (Gao et al., 2021a; Gao et al., 2021b; Goodall and Wickramasinghe, 2021).

Metastasis-associated lung adenocarcinoma transcript 1 (MALAT1) was initially found to regulate cancer metastasis. The RNA sequencing profile used to analyze lncRNA expression showed that MALAT1 was significantly upregulated in HUVECs treated with hypoxia (Michalik et al., 2014). Silencing of MALAT1 increased basal endothelial cell migration and sprouting *in vitro*. MALAT1 can activate PI3K and Akt phosphorylation, promoting endothelial apoptosis by sponging miR-126 (Zhang et al., 2020a). Furthermore, MALAT1 can also protect the angiogenesis function through the miR-205-5p/VEGFA pathway (Gao et al., 2020). Microarray data showed that the expression of lncRNA taurine upregulated gene 1 (TUG1) was associated with the ox-LDL concentration. Runt-related transcription factor 2 (Runx2) was detected as the downstream target of TUG1 by RNA pull down, and Runx2 knockdown could reverse the function of aminopeptidase N (ANPEP). TUG1 silencing can promote endothelial injury repair *via* the repression of Runx2 and ANPEP (Du et al., 2021).

The expression of lncRNA plasmacytoma variant translocation 1 (PVT1) was upregulated in ox-LDL-induced injury in HUVECs. PVT1 knockdown decreased the inflammation and apoptosis in HUVEC *via* the miR-153-3p/GRB2 axis (Guo et al., 2021). Gao et al. reported that lncRNA H19 could regulate ox-LDL inflammation, apoptosis and HUVECs by targeting the miR-let-7/periostin axis (Cao et al., 2019). The expression level of lncRNA differentiation antagonizing non-protein coding RNA (DANCER) was increased in patients with atherosclerosis and ox-LDL-treated cells. DANCER knockdown significantly reduced the levels of IL-6 and, TNF-α *via* miR-214-5p sponging, thereby activating the chaperone of cytochrome c oxidase subunit II COX2 (COX20) (Zhang et al., 2022). The down-regulated expression of long intergenic non-coding 00299 (LINC00299) inhibited vascular injury through the miR-135a-5p/XBP1 axis (Chang et al., 2022). The expression level of lncRNA ZEB1-antisense 1 (ZEB1-AS1) was increased in the ox-LDL induced endothelial cell injury model. Nucleotide-binding oligomerization domain 2 (NOD2) was reported to integrate ER stress and innate immunity. ZEB1-AS1 expression can regulate endothelial cell injury *via* LRPPRC to stabilize NOD2 mRNA (Xu et al., 2019b). The expression level of lncRNA OIP5-AS1 was increased in ox-LDL mediated vascular ED. Zhang et al. demonstrated that OIP5-AS1 knockdown suppressed apoptosis and evaluated cell viability by modulating the expression of miR-320a and regulation of lectin-like oxidized low-density lipoprotein receptor 1 (Zhang et al., 2020b).

## 4 Conclusion

Diabetic patients have extensive vascular disease. Previous epidemiological data and laboratory mechanism studies have indicated that vascular complications are the leading cause of morbidity and mortality in T2D. During the development of the disease, its damage to blood vessels includes increased thickness of blood vessels, decreased vasodilation, vascular calcification, and impaired vascular response. Awareness of T2D-related diseases and complications, identification of their possible pathogeneses and causes, and development of new interventions are warranted. Recent studies have shown increasing interest in identifying the mechanisms that play a major role in the development of T2D. We reviewed and discussed the roles of ED, inflammation, oxidative stress and epigenetic modification in the pathogenesis of T2D induced by hyperglycemia and hyperlipidemia.

The early symptom of diabetic vascular disease is endothelial damage, which can cause significant dysfunction. The formation of thrombosis and activation of inflammation are the main features of vascular injury, which can decrease vasodilation. The inflammatory process contributes to insulin resistance, and long-term chronic inflammation can further cause endothelial damage and exacerbate oxidative stress. Oxidative stress is mainly due to mitochondria dysfunction. Mitochondria are energy-producing factories, and mitochondrial dysfunction can induce apoptosis and activate inflammation, creating a vicious cycle. In the pathological process of T2D, dysregulation of DNA methylation, histone markers and non-coding RNA activate dormant endothelial cells and initiates various molecular activities, resulting in ED. The interaction between the factors that cause vascular injury is inseparable. ED is the first event to occur and the ultimate point of action of elements.

Continuous experimental, clinical and translational studies have shown that pharmacological interventions targeting glycotoxicity and lipotoxicity play an important role in the treatment of the vascular complications of T2D. Most of these drugs have shown good vascular protection in preclinical and clinical studies. For instance, the common drugs used to treat diabetes, such as insulin and metformin, have been shown to be effective in stimulating the release of NO and maintaining vascular stability (Tousoulis et al., 2008; Aggarwal et al., 2021). Moreover, metformin can regulate AMPK-related pathways to protect endothelial cells (Ding et al., 2021). Sodium-glucose cotransporter 2 inhibitors (SGLT2i) are new-generation drugs for the treatment of T2D and its complications (DeFronzo et al., 2021). The mechanisms of action of SGLT2i include anti-inflammatory, anti-proliferative, and antifibrotic effects (Heerspink et al., 2019; DeFronzo et al., 2021). The protective effects of GLP-1 receptor agonists mainly include maintaining the integrity of the intima, reducing the adhesion of monocytes stimulated by ox-LDL, increasing the level of NO production, reducing the release of inflammatory factor, and blocking inflammatory pathways (Chang et al., 2019; Xu et al., 2019c). Moreover, statins, which are lipid-lowering drugs, can reduce LDL content and increase the bioavailability of NO. They have anti-apoptotic and anti-inflammatory effects, which reduce the release of inflammatory factors and inhibit leukocyte adhesion. Antihypertensive drugs such as calcium channel blockers (CCBs), ARBs, and ACEI can also improve the endothelial function and increase the eNOS expression level (Silva et al., 2019). Other studies suggested that ACEI, and, ARBs could also attenuate ROS-induced injury by enhancing the activity of superoxide dismutase. Anti-inflammatory drugs, including nonselective (aspirin) and selective COX-2 inhibitors, have been studied to reduce cardiovascular risk and recurrence of cardiovascular events in patients with T2D (Huang and Vita, 2006). Some natural ingredients such as colchicine have been used clinically for the treatment of ED and cardiovascular disease. *Angelica sinensis* polysaccharide, which is purified from the fresh roots of *Angelica sinensis,* could regulate glucose and lipid metabolisms by reducing the release of inflammatory factors (Wang et al., 2015). Lifestyle changes, including diet and exercise, are also the primary recommendations. The mechanism of action of exercise therapy involves promoting the release of cytokines and increasing the uptake and utilization of glucose and lipid hydrolysis (Yang et al., 2019b).

By better understanding the etiology of vascular diseases, combining the relevant mechanisms of new and old drugs, and developing targeted interventions for the vascular complications of diabetes, cardiovascular morbidity and mortality rates in patients with T2D can be reduced.
